# The Patient Activation Measure (PAM) and the pandemic: Predictors of patient activation among Australian health consumers during the COVID‐19 pandemic

**DOI:** 10.1111/hex.13725

**Published:** 2023-02-21

**Authors:** Genevieve Dammery, Kathryn Vitangcol, James Ansell, Louise A. Ellis, Carolynn L. Smith, Ann Carrigan, Jeffrey Braithwaite, Yvonne Zurynski

**Affiliations:** ^1^ Centre for Healthcare Resilience and Implementation Science, Australian Institute of Health Innovation Macquarie University Sydney Australia; ^2^ NHMRC Partnership Centre for Health System Sustainability Macquarie University Sydney Australia; ^3^ Centre for Health Services Research The University of Queensland Woolloongabba Queensland Australia; ^4^ Consumers Health Forum of Australia Deakin West Australian Capital Territory Australia

**Keywords:** activation, care delivery, COVID‐19, mental health, Patient Activation Measure, self‐care

## Abstract

**Background:**

Preventative healthcare is crucial for improving individual patient outcomes and is integral to sustainable health systems. The effectiveness of prevention programs is enhanced by activated populations who are capable of managing their own health and are proactive to keep themselves well. However, little is known about the level of activation among people drawn from general populations. We used the Patient Activation Measure (PAM) to address this knowledge gap.

**Methods:**

A representative, population‐based survey of Australian adults was conducted in October 2021 during the Delta strain outbreak of the COVID‐19 pandemic. Comprehensive demographic information was collected, and the participants completed the Kessler‐6 psychological distress scale (K6) and PAM. Multinomial and binomial logistic regression analyses were performed to determine the effect of demographic factors on PAM scores, which are categorised into four levels: 1—participants disengaged with their health; 2—becoming aware of how to manage their health; 3—acting on their health; and 4—engaging with preventative healthcare and advocating for themselves.

**Results:**

Of 5100 participants, 7.8% scored at PAM level 1; 13.7% level 2, 45.3% level 3, and 33.2% level 4. The mean score was 66.1, corresponding to PAM level 3. More than half of the participants (59.2%) reported having one or more chronic conditions. Respondents aged 18 to 24 years old were twice as likely to score PAM level 1 compared with people aged 25–44 (*p* < .001) or people aged over 65 years (*p* < .05). Speaking a language other than English at home was significantly associated with having low PAM (*p* < .05). Greater psychological distress scores (K6) were significantly predictive of low PAM scores (*p* < .001).

**Conclusion:**

Overall, Australian adults showed high levels of patient activation in 2021. People with lower incomes, of younger age, and those experiencing psychological distress were more likely to have low activation. Understanding the level of activation enables targeting sociodemographic groups for extra support to increase the capacity to engage in prevention activities. Conducted during the COVID‐19 pandemic, our study provides a baseline for comparison as we move out of the pandemic and associated restrictions and lockdowns.

**Patient or Public Contribution:**

The study and survey questions were co‐designed with consumer researchers from the Consumers Health Forum of Australia (CHF) as equal partners. Researchers from CHF were involved in the analysis of data and production of all publications using data from the consumer sentiment survey.

## 1 INTRODUCTION

The ability of people to look after their own health, feel confident in accessing medical treatment, and effectively engage in preventative healthcare is crucial to maintaining healthcare quality and safety and achieving improved patient outcomes.^1^, ^2^ There is an increasing call for consumer‐led care, and to embrace codesign with patients at all levels, from management boards to the clinics at the frontlines of care.^3^ However, to ensure effective involvement from consumers, it is important that they feel empowered and have the capacity to take an active role in their own healthcare, while effectively and confidently engaging with healthcare services. The COVID‐19 pandemic challenged and tested the capacity of populations to proactively seek, engage with, and follow health advice to keep themselves and others safe from infection. At the same time, the population had to cope with lockdown and movement restrictions for long periods of time (over 100 days in some cases).^4^ However, little is known about the capacity of populations for self‐care during crises such as the COVID‐19 pandemic.

First developed in 2005, the Patient Activation Measure (PAM) is a validated tool to measure patient activation, which is defined as ‘an individual's knowledge, skill, and confidence for managing their health and health care’.^5^

Patients with higher levels of activation take a greater active role in self‐management of their health and are more likely to seek health information, leading to better patient outcomes and better experiences with the healthcare system.^1^ Low activation, on the other hand, is generally found in patients with chronic conditions and those with worse self‐reported health status.^6^ Patient activation has been linked to a person's level of health literacy, and whilst there has been research showing that patient activation tends to be lower in older populations and in those with chronic conditions,^1^, ^7^, ^8^ there is a limited body of research on what other predictors exist for patient activation within the general population. In previous research, the PAM has been found to be a reliable predictor of medication adherence,^9^ engagement with prevention strategies, reductions in unnecessary emergency department (ED) visits,^10^ and hospital readmission rates.^10^ Studies have shown that a single point increase in PAM score equates to a 2%–3% gain in health outcomes and a decrease in health service use.^2^, ^11^

It is estimated that healthcare for people with chronic conditions costs the Australian health system AU$38 billion per year,^12^ and nearly AU$5.4 trillion in the United States.^13^ Given the evidence that chronic disease prevention and public health interventions can reduce healthcare expenditure,^14^, ^15^ it is crucial that measures are taken to understand activation among patients with and without chronic disease and their capacity to self‐manage their health.

In addition to the capacity for people to protect themselves from COVID‐19 infection, the capacity and motivation for self‐management of health conditions were recognised as particularly important during the pandemic, when routine access to health services for the detection, monitoring and management of health conditions was reduced.^16^ During the initial wave of the pandemic in 2020, it was found that people with mental distress were six times more likely to avoid necessary healthcare than those without, as were people aged between 18 and 44 years, and those under financial stress.^17^ With people avoiding care^17^ and the global health system being over‐burdened ^18^, ^19^ due to the pandemic, it is important to know how many individuals have the skills and willingness to engage with the healthcare system to self‐manage their health.

This study aimed to address a current gap in knowledge about the level of activation among the general population and to better understand the predictors for patient activation in a representative sample of Australians recruited during the second year of the COVID‐19 pandemic.

## 2 METHODS

### 2.1 Participant recruitment

Australian participants aged ≥18 years were recruited through Dynata, an international market research company that conducts over 100 million surveys annually. Panellists that are registered with Dynata opt‐in to participate in online research in exchange for a reward of cash or points. The survey was conducted over a 2‐week period in October 2021. Ethics approval was granted by The Macquarie University Human Research Ethics Committee (Ref no: 52021367031878). Participants were contacted via email by Dynata and invited to participate, giving informed consent when they opted in to take part in the survey. Deliberate oversampling of people that identified as Aboriginal and/or Torres Strait Islanders and those living in rural and remote parts of Australia ensured that there was a large enough cohort for accurate comparisons to be made.

### 2.2 Survey design

The survey was co‐designed with researchers from the Australian Institute of Health Innovation and consumer researchers from the Consumers Health Forum of Australia (CHF), with additional feedback provided by the Australian Government Department of Health. Survey questions were consistent with those asked in the 2018 Australian Consumer Sentiment Survey.^20^, ^21^ Several additional questions were included in the 2021 iteration: questions of perceptions of the Australian healthcare system during the COVID‐19 pandemic, and whether affordability and access to care had been impacted by the pandemic. Further, the Patient Activation Measure‐10 (PAM‐10)^5^ and Kessler‐6 Psychological distress measure (K6)^22^ were added to the survey in 2021. The survey contained 67 items and took 30–40 min to complete. Survey questions were piloted by the research team before distribution.

### 2.3 PAM

The PAM‐10 is a validated tool that is used to assess the extent to which an individual can self‐manage their health. Respondents rated their level of agreement with 10 statements about their knowledge, confidence, and skills to understand their health on a 5‐point Likert scale (Totally Disagree, Disagree, Agree, Totally Agree, or Not Applicable (N/A)) (Appendix A). The PAM is comprised of items that are scored on a scale of 0‐100, dividing participants into four activation levels:

1. Level 1: Individuals tend to be passive and feel overwhelmed by managing their own health. They may not understand their role in the care process.
2. Level 2: Individuals may lack the knowledge and confidence to manage their health.
3. Level 3: Individuals appear to be taking action but may still lack the confidence and skill to support their behaviours.
4. Level 4: Individuals have adopted many of the behaviours needed to support their health but may not be able to maintain them in the face of life stressors.^23^

Responses to the PAM were scored by Insignia Health, the company that licenses the PAM. Scores ranged from 0 (indicative of low activation) to 100 (indicative of high activation) and were then categorised into four levels. The cut‐offs for the four PAM levels are: 1—score of 0.0–47.0, 2—score of 47.1–55.1, 3—score of 55.2–72.4, and 4—score of 72.5–100.

### 2.4 Mental health status

Mental health status was assessed using the K6 which is a validated tool that measures levels of psychological distress in an individual.^24^ It consists of six questions that ask the respondent about the frequency of feelings of sadness, nervousness, restlessness, hopelessness, worthlessness and feeling that everything is an effort during the past month, on a scale of 1–5 (none of the time to all of the time).^22^ The K6 can be used to accurately predict mental illness in an adult population.^22^ Internal consistency of the K6 items was high in the overall sample of 5100 respondents (Cronbach's *α* = .94; 95% confidence interval [CI]: 0.93–0.95) with the six items loading primarily on one factor.^25^ For the analysis of the K6 in this paper and in line with the Australian Bureau of Statistics,^26^ scoring was dichotomous, with scores of 6–18 indicating ‘no probable serious mental illness’, and scores of 19‐30 indicating ‘probable serious mental illness’.^26^

### 2.5 Income status

Weekly household income was classified into a dichotomous variable for analysis: earning < $2000 per week or earning > $2000 per week. Income status coding was based on the average household weekly income as measured by the Australian Bureau of Statistics (ABS) in 2020: $2086.^27^

### 2.6 Chronic conditions

Survey participants were asked if they lived with any of the 10 chronic conditions that are most commonly reported according to the Australian Institute of Health and Welfare (AIHW): arthritis, asthma, back pain, cancer, cardiovascular disease, chronic obstructive pulmonary disease, diabetes, chronic kidney disease, mental health conditions, and osteoporosis.^24^ Participants were also given the opportunity to list other chronic diseases that they may have been diagnosed with, or they could select ‘none of the above’.

### 2.7 Cultural background

The cultural background of participants was assessed in two questions: ‘Are you of Aboriginal and/or Torres Strait Islander origin?’ and ‘Do you speak a language other than English at home?’. Asking about respondents' cultural background assists in understanding what barriers exist when accessing and affording care, and is important as a step in eliminating these disparities.^28^ We also sought to understand whether culturally specific demographic factors were associated with activation for self‐care.

### 2.8 Geographical location

The geographical location of respondents was ascertained by collecting individual's postcodes. Postcode data was then used to categorise individuals according to the Accessibility/Remoteness Index of Australia (ARIA+): major cities, inner regional, outer regional, remote, or very remote. For analysis, these categories were recoded into a dichotomous variable: major cities or regional/remote areas. It is widely reported that there are substantial barriers to accessing healthcare in regional and remote areas, both in Australia and internationally, resulting in poorer outcomes for those individuals.^29^, ^30^ The survey was conducted during the peak of the COVID‐19 delta outbreak in Australia, leading to severe lockdowns lasting over 100 days in two Australian states: New South Wales (NSW) and Victoria (VIC). As such, the geographical location was also coded dichotomously as ‘NSW or Victoria’ and ‘all other states’.

### 2.9 Private health insurance status

Respondents were asked whether they had private insurance membership and were asked to provide a reason for why or why not, depending on their response. In Australia, private health insurance status has been identified as having a relationship with socioeconomic status^31^, ^32^ and health literacy levels.^33^ We sought to understand whether there was a relationship between private health insurance status and patient activation.

### 2.10 Statistical analysis

Minor postweighting adjustments by gender, age and state were made in accordance with our previous survey analysis^20^, ^21^ to reflect population distribution according to the Australian Bureau of Statistics demographic statistics of March 2021.^34^ Weighting adjustments were performed using the anesrake package in R,^35^ and descriptive and inferential statistical analysis was conducted using IBM SPSS Statistics V27.^36^

A multinomial logistic regression analysis was used to investigate the effect of gender, age, income, speaking a language other than English at home, Aboriginal and/or Torres Strait Islander status, education level, private health insurance status, and chronic condition status on the four PAM levels. Binomial logistic regression was performed for comparing PAM level 1 versus levels 2–4, PAM level 4 versus levels 1–3, and PAM levels 1 and 2 versus 3 and 4.

A hierarchical multiple regression model was further developed to assess the contribution of the K6 score to predict patient activation. Linear correlation analyses were performed to determine predictor significance and order of entry into the regression model. Demographic characteristics of gender, age, income, speaking a language other than English at home, Aboriginal and/or Torres Strait Islander status, education level, private health insurance status, and chronic condition status were controlled for in stage one. K6 was entered as the independent variable in stage two. Statistical significance was considered at *p* < .05.

## 3 RESULTS

### 3.1 Overview of the sample

The sample consisted of 5100 Australians aged between 18 and 92 years (*M* = 45.7, SD = 17.6) recruited based on representative quotas for geographical location (metropolitan vs rural/remote) and population size in each of the Australian jurisdictions (states and territories), age and gender, based on 2021 ABS data (Table 1).^37^ Due to the nature of participant recruitment, the survey response rate could not be calculated. The internal consistency for the PAM‐10 responses in our survey was calculated using IBM SPSS Statistics V27,^36^ and was deemed to have a good level of consistency (*α* = .84).

**Table 1:** Demographic characteristics of survey respondents.

Number of respondents (*N*)	5100
**Characteristics**	*N* a (%)^b^
Gender	
Male	2475 (48.7)
Female	2576 (50.7)
Non‐binary	26 (0.6)
Age group	
18–24	614 (12.0)
25–44	1853 (36.3)
45–54	1589 (31.2)
65+	1043 (20.5)
State	
New South Wales (NSW)	1623 (31.8)
Victoria (Vic)	1319 (25.9)
Queensland (Qld)	1033 (20.3)
South Australia (SA)	351 (6.9)
Western Australia (WA)	531 (10.4)
Tasmania (Tas)	108 (2.1)
Northern Territory (NT)	49 (1.0)
Australian Capital Territory (ACT)	86 (1.7)
Major city	2980 (58.4)
Regional/remote	2120 (41.6)
Identifies as Aboriginal and/or Torres Strait Islander	586 (11.5)
Speaks a language other than English at home	1251 (24.5)

^a^ Unweighted.

b Weighted.

### 3.2 Chronic condition status

Of the 5100 people surveyed, 3021 (59.2%) reported living with one or more chronic conditions. The most commonly reported chronic condition was back pain or back problems, (Table 2). These proportions are consistent with the healthcare consumer sentiment survey delivered in 2018.^20^, ^21^

**Table 2:** Chronic condition status in survey respondents.

Condition reported	Number (%)
Arthritis	989 (19.4)
Asthma	981 (16.3)
Back pain or back problems	1266 (24.8)
Cancers	246 (4.8)
Cardiovascular disease	373 (7.3)
Chronic obstructive pulmonary disease	203 (4.0)
Diabetes	581 (11.4)
Kidney disease	130 (2.5)
Mental disorders	851 (16.7)
Osteoporosis	186 (3.6)

### 3.3 Respondents who identified as Aboriginal and/or Torres Strait Islander

A subset of our sample identified as Aboriginal and/or Torres Strait Islander (*n* = 586, 11.5%). Our analysis showed that 55.4% of people identifying as Aboriginal and/or Torres Strait Islanders received a household income > $2000 per week, compared to 31.5% of the general population. Almost half (45.7%) of people identifying as Aboriginal and/or Torres Strait Islanders lived in either metropolitan Sydney or Melbourne, and 62.3% overall lived in metropolitan areas. Of the 586 respondents that identified as Aboriginal and/or Torres Strait Islander, most reported having at least one chronic condition (*n* = 519, 89.0%).

### 3.4 Distribution of PAM and K6 scores

A K6 score could be calculated for 5,034 respondents, representing 98.7% of surveyed individuals. Incomplete survey responses or responses considered unreliable during scoring were not included. Almost one‐quarter (23.6%, *n* = 1203) of respondents scored in the ‘serious psychological distress’ range of the K6.

The mean patient activation score was 66.1 (SD = 19.07), corresponding to level 3 activation. Overall, respondents showed high levels of patient activation with 78.5% reporting level 3 or 4 activation, while over 20% scored at level 2 or one, or low activation (Table 3).

**Table 3:** PAM activation level.

	Frequency	Percent
Level 1	393	7.8
Level 2	692	13.7
Level 3	2279	45.3
Level 4	1670	33.2
Total	5034	

Abbreviation: PAM, Patient Activation Measure.

### 3.5 Predictors for patient activation

Likelihood ratio tests revealed that gender, age, income, speaking a language other than English at home, identifying as Aboriginal and/or Torres Strait Islander, having one or more chronic conditions, and private health insurance status had a significant overall effect on PAM.

Relative to those with PAM levels 2–4, those who reported speaking a language other than English were 57.5% more likely to have a PAM level 1 score than those who spoke only English at home (*b* = −0.515, *p* < .01). Respondents who had no private health insurance were 84.5% more likely to have a PAM level 1 score than those with insurance (*b* = 0.591, *p* < .001). People aged 18–24 were twice as likely to have PAM level 1 (*b* = 0.809, *p* < .001) compared with people aged 25–44. People aged 65 years or more were significantly less likely to have a PAM level 1 score than those aged 25–44 (57.3%; *b* = −0.557 *p* < .05). Respondents who did not have a chronic condition were twice as likely to have a PAM level 1 score than those with a chronic condition (*b* = −0.774, *p* < .001) (Table 4).

**Table 4:** Relationship between PAM level 1 and demographic data relative to PAM levels 2–4.

PAM activation level	*B*	SE	Odds ratio
*Level 1*			
Education: university and above	−0.100	0.138	0.905
Speaks a language other than English at home	−0.515	0.143	0.597*
Aboriginal and/or Torres Strait Islander	−0.214	0.168	0.807
Has a chronic condition	−0.774	0.143	0.461***
Has private health insurance	0.591	0.136	0.542***
Income: >$2000/week	0.185	0.138	1.03
Lives in NSW/VIC	0.103	0.123	1.108
Regional	0.140	0.130	1.150
Age: 18–24	0.809	0.162	2.245***
Age: 45–64	−0.191	0.154	0.826
Age: 65+	−0.557	0.206	0.573*
Female	−0.054	0.123	0.948

*Note*: Reference category for the PAM level is all other levels. Reference categories for the demographics are education level below university, speaks only English, not Aboriginal and/or Torres Strait Islander, no chronic condition, no private health insurance, income < $2000/week, lives in all other states, metro region, age 25–44, and male.

Abbreviation: PAM, Patient Activation Measure.

*p < .05; ***p < .001.

The hierarchical multiple regression revealed that at stage one, demographics significantly contributed to the regression model (*F* = 11.152, *p* < .001) and accounted for 2.6% of the variation in PAM scores. Adding the K6 score explained an additional 3.6% of the variation in the PAM score, and this change in *R* ^2^ was significant (*F* = 14.251, *p* < .001). When all independent variables were included in the final stage of the regression model, the K6 score was a significant predictor of PAM score. For each 1‐point increase in K6 scores, there was a decrease in the PAM score by 0.127 (*p* < .001). Of note, the final model also showed that factors such as being aged between 18 and 24 or speaking a language other than English were significantly predictive of low patient activation, decreasing scores by 0.043 (*p* < .01) and 0.042 (*p* < .05) respectively. Having private health insurance and higher income was also significantly associated with PAM, increasing PAM scores by 0.056 (*p* < .001) and 0.090 (*p* < .001), respectively. Finally, identifying as Aboriginal and/or Torres Strait Islander or identifying as female were also significantly associated with PAM, increasing PAM scores by 0.090 (*p* < .001) and 0.035 (*p* < .05), respectively (Table 5).

**Table 5:** Predictors of PAM: Hierarchical multiple regression.

	**Stage 1**			**Stage 2**		
**Variable** a	**Unstandardised beta (*B*)**	**Standard error for unstandardised beta (SE)**	**Standardised beta (*β*)**	**Unstandardised beta (*B*)**	**Standard error for unstandardised beta (SE)**	**Standardised beta (*β*)**
Age: 18–24	−2.960	0.915	−.052**	−2.423	0.913	−.043**
Age: 45–64	1.578	0.648	.042**	0.496	0.663	.013
Age: 65+	3.182	0.788	.073***	0.978	0.844	.023
Education: university and above	0.613	0.596	.017	0.819	0.593	.023
Has private health	1.953	0.576	.054***	1.998	0.573	.056***
Aboriginal and/or Torres Strait Islander	3.737	0.879	.070***	4.831	0.888	.090***
Has a chronic condition	−2.176	0.552	−.060***	−0.863	0.579	−.024
Speaks a language other than English at home	−1.918	0.684	−.047**	−1.716	0.681	−.042*
Regional	0.603	0.548	.017	0.703	0.546	.019
Lives in NSW/VIC	−0.942	0.531	−.026	−0.771	0.529	−.022
Income > $2000/week	3.460	0.600	.094***	3.321	0.597	.090***
Female	1.187	0.538	.034*	1.215	0.535	.035*
K6 score	‐			−0.325	0.046	−.127***
Adjusted *R* ^2^	0.026			0.036		
*F*	11.152			14.251		
Δ*R* ^2^ b				0.010		
Δ*F* c				3.099		

Abbreviation: PAM, Patient Activation Measure.

^a^ Reference categories: age 25–44 years, education below university level, no private health insurance, no chronic condition, speaks only English, metro region, all other states, income < $2000/week, and male.

b Δ*R* ^2^ is the incremental increase in the model *R* ^2^ (measure of the proportion of variability) resulting from the addition of a predictor, or set of predictors, to the regression equation.

c ΔF is the change in the *F*‐statistic (value for general significance of a set of explanatory variables in regression analysis).

* *p* < .05

** *p* < .01

*** *p* < .001.

## 4 DISCUSSION

This study provides unique data about the level of activation in a representative sample of Australian adults surveyed during the second year of the COVID‐19 pandemic (2021). More than 75% of respondents had high levels of patient activation (PAM levels 3 and 4), suggesting that a majority of respondents believed they had the skills and knowledge to look after their health. Factors such as higher household income, older age, having a chronic condition and being female influenced having a higher activation level. People who spoke a language other than English at home had lower activation levels. Previous research has suggested that the ability to communicate with healthcare professionals is important in helping people to engage with the healthcare system,^38^ and as such, strategies to reach such communities to better engage with their own healthcare is imperative, especially in culturally and linguistically diverse communities throughout Australia and elsewhere. Such strategies need to be developed with communities as central partners, taking into account their current capabilities, needs, wants and contexts, to ensure preparedness for future health crises, including pandemics.

Respondents who had higher levels of psychological distress on the K6 scale reported low levels of activation (PAM 1 or 2). This suggests that individuals with poorer mental health and psychological distress are more likely to have impaired capacity for self‐management of their health and health care. Whilst there is a correlation between higher levels of psychological distress and lower PAM scores, the causality of this relationship is unclear, that is, it is unclear whether psychological distress contributes to low activation or vice versa. It is understood that patients with higher activation have more collaborative relationships with their healthcare providers.^34^ The paradigm of healthcare delivery remains, in some places, one of professionally‐dominated decision‐making (so‐called ‘paternalism’) rather than one where more emphasis is placed on respecting patient determination, and deploying shared decision‐making models, although this is changing. Improving patient activation widely, and especially amongst people with poor mental health and other chronic conditions can be hindered by a perpetuation of traditional delivery models.^39^ However, with an increasingly greater focus on person‐centred care delivery, many providers have risen to the challenge and exploited the potential to harness patient activation to improve health outcomes.^40^, ^41^ It is critical for health care providers to factor into their treatment plans or prevention strategies, knowledge about the patient's determinants of activation, including understanding their level of psychological distress, their age, and socioeconomic challenges including their ability to pay for needed care in the absence of private health insurance cover.

In 2019, the CHF used the PAM to survey 1703 people who reported having one or more chronic conditions. This survey found that despite having a chronic condition, most of those surveyed had relatively high activation levels, with 41% scoring at level 3 activation and 27% having level 4 activation.^42^ Our 2021 results are in alignment with the CHF study, with 43% of people with chronic condition scoring PAM level 3, and 31% PAM level 4 in our survey. The reasons for the relatively high activation among people with chronic conditions are uncertain and should be further investigated through qualitative research. However, it is noteworthy that having one or more chronic conditions was significantly associated with a greater likelihood of low activation (PAM level 1 or 2) compared with people who did not have chronic conditions, suggesting that the level of patient activation is also an important factor to consider when providing health information, health advice or treating people with chronic conditions. Ultimately, the results of the 2021 survey suggest that the COVID‐19 pandemic did not dramatically change the average activation levels among people living with chronic conditions in Australia, possibly due to the fact that people with chronic conditions are used to managing their health.

### 4.1 Interventions to increase patient activation

It has been suggested that non‐medical peer support interventions that are tailored to an individual's particular needs lead to improved health outcomes and encourage patients to continue engaging with the recommended intervention.^40^ Social prescribing, which often includes these components, involves referring patients to non‐clinical services, such as social programs, healthy lifestyle programs or physical activity programs with the aim of addressing the social determinants of health.^41^ Co‐design is a central tenet of social prescribing, whereby the patient and a healthcare professional, known as a link worker, co‐produce an action plan. This increases the likelihood that the patient will engage with the service or intervention,^40^ and frequently results in both improved activation levels and health outcomes.^40^ Although the body of literature is limited in this area, studies have demonstrated the benefits of social prescribing in improving activation in patients with both lower and higher levels of activation.^43^ Challenges may arise where patients with lower activation are less likely to engage in programs and interventions aimed at increasing their activation,^23^ leaving the responsibility to their healthcare providers to attempt to increase activation whilst providing routine care. Fortunately, there is evidence to suggest that patient activation can be increased by the behaviour of primary care clinicians.^44^ Clinicians who are more supportive of patient self‐management are more likely to engage with patients, which leads to partnership‐building and more collaborative behaviours, and as a result, increases patient activation levels. By engaging and activating patients, their health outcomes improve through supported self‐management.^45^

### 4.2 Implications

Our study highlights that most Australian healthcare consumers have high levels of activation and are capable of participating in the management of their health. However, 2 in 10 respondents reported low activation, including people with mental health conditions, those with high psychological distress and vulnerable groups such as people living with financial stress and culturally and linguistically diverse groups. Further developing a strong organisational emphasis on the value and delivery of person‐centred care will require training for healthcare professionals to raise awareness that every patient encounter is an opportunity for person‐centred care and for increasing patient activation,^46^ especially among identified vulnerable groups. Delivering person‐centred care and empowering patients requires shared decision‐making, the provision of information appropriate to patients' care needs and validation of their expectations and experiences, thereby enabling patients to actively engage in their treatment and to better understand their health condition. Healthcare professionals who have not yet changed their practice or have been slow to adopt shared decision‐making models of care should be more cognizant of the importance of empowering their patients to take control of their health by collaboratively setting goals and discussing their healthcare.

This study provides a valuable point of comparison for future studies. The COVID‐19 pandemic has had far‐reaching consequences for human health beyond the direct impact of becoming infected with the virus. Extended lockdowns were consistently demonstrated to negatively influence mental health,^47^ and chronic condition management was impacted as a result of limited access to care delivered by health professionals.^48^ Furthermore, the implications of pausing routine screening tests,^49^ and elective surgeries are not yet fully realised. With poor mental health and inability to access healthcare services having been shown to lower patient activation,^50^ it is critically important to know which individuals are more impacted by these factors so that resources can be directed to assist them, and so they can be empowered to engage with self‐care.

### 4.3 Strengths and limitations

A strength of this study is the representative sample of the Australian population, which ensures that our data accurately reflect the views or behaviours that we sought to better understand, while also allowing for a high level of accuracy and minimising biases that can arise from sampling errors.

This study is further strengthened by the co‐design of the survey with the Australian Institute of Health Innovation, the CHF, and the Australian Government Department of Health. This enabled identification of relevant questions, the credibility of the knowledge produced, and application of results to be adapted to various contexts, while simultaneously permitting the survey questions to be user‐friendly, concrete, specific and relevant for participants. As such, the co‐designed approach ultimately aided in accessing participants, improving response rates, and recruitment from seldom‐heard groups.

Aboriginal and Torres Strait Islander peoples account for 3%–4% of the Australian population,^51^ and surveys such as ours often result in small samples for which data are difficult to interpret. Despite the purposeful oversampling of the Aboriginal and Torres Strait Islander population in our survey and collecting a substantial sample of 587 responses for this group, the sample may remain unrepresentative. In our survey, incomes reported by Aboriginal and/or Torres Strait Islander populations were higher than those reported by the AIHW.^52^ AIHW data consistently indicates that the median household income for those who identified as Aboriginal and/or Torres Strait Islander is lower compared to the non‐Indigenous population.^52^ For consistency with our previous publications, data was post‐weighted by gender, age, and state to reflect the Australian population distribution according to the ABS demographic statistics of March 2021.^34^ This ABS data set does not provide an analysis of the Australian population by Indigenous status and income, and thus in this study we did not weight by these variables. As a result, our weighting procedure does not negate oversampling of Aboriginal and/or Torres Strait Islanders in this case, potentially leading to an overestimation of income among the sample of Aboriginal and/or Torres Strait Islander people included in this study. The geographical location for those identifying as Aboriginal and/or Torres Strait Islanders were also vastly concentrated in metropolitan cities, at 62%, compared to the national average of 38% of people identifying as Aboriginal and/or Torres Strait Islander living in metropolitan cities according to the ABS^53^ and AIHW.^54^ For this reason, we have not reported comparison results, instead reporting on the whole sample of participants. This sampling bias in recruiting people who identify as Aboriginal and/or Torres Strait Islander is likely a result of recruiting participants via the Internet from an existing market research panel and indicates the need for different recruitment strategies to be undertaken in future surveys. As participants were recruited through an established survey panel, the response rate could not be calculated. Furthermore, our participants are individuals that have sought participation in incentivised research, and therefore may not be truly representative of the whole Australian population.

Our survey was distributed during the COVID‐19 pandemic when the majority of the population of the states of NSW and Victoria were in lockdown due to the second wave of the virus. As current evidence demonstrates, lockdowns contribute to poor mental health outcomes,^55^ and the potential impact of the timing of the survey on reported K6 and PAM scores should be noted. Nevertheless, this survey represents a pandemic baseline of mental distress and patient activation levels and will serve as a comparison data set to track how the population recovers as strict lockdowns are lifted, and the pandemic wanes. In addition, the findings are based on a cross‐sectional analysis, limiting our understanding of the temporal ordering of circumstances and behaviours.

## 5 CONCLUSIONS

Our nationally representative survey of Australian adults during the COVID‐19 pandemic revealed that patient activation was lower in distressed populations with lower income, who did not have private health insurance and spoke a language other than English at home. As the COVID‐19 pandemic continues to evolve, patients' capacities to manage their health and the negative repercussions of the pandemic continue, targeted support is needed to help people maintain mental and physical health. Furthermore, the impact of the pandemic on the PAM will become apparent as future population‐based surveys are deployed in Australia. Our findings extend current knowledge of patient activation levels in Australia and identify groups who would benefit from interventions to improve patient activation, thereby improving health outcomes and reducing the burden on the healthcare system from chronic disease care. Programs that focus on specific skills, such as problem‐solving and resource utilisation, may be an important approach to support self‐management, especially if done in conjunction with primary healthcare providers.

## CONFLICT OF INTEREST STATEMENT

The authors declare no conflict of interest.

## Data Availability

The data that support the findings of this study are available from the corresponding author upon reasonable request.

## APPENDIX A

See Table A1

**Table A1:** Statements listed in the Patient Activation Measure questionnaire.

1. When all is said and done, I am the person who is responsible for taking care of my health. 2. Taking an active role in my own health care is the most important thing that affects my health. 3. I know what each of my prescribed medications do. 4. I am confident that I can tell whether I need to go to the doctor or whether I can take care of a health problem myself. 5. I am confident that I can tell a doctor concerns I have even when he or she does not ask. 6. I am confident that I can follow through on medical treatments I may need to do at home. 7. I have been able to maintain (keep up with) lifestyle changes, like eating right or exercising. 8. I know how to prevent problems with my health. 9. I am confident I can figure out solutions when new problems arise with my health. 10. I am confident that I can maintain lifestyle changes, like eating right and exercising, even during times of stress.
